# Screening and validation of atherosclerosis PAN-apoptotic immune-related genes based on single-cell sequencing

**DOI:** 10.3389/fimmu.2024.1297298

**Published:** 2024-04-26

**Authors:** Yamin Song, Bo Lou, Huiting Wang, Guifeng Zhang, Yitong Xia, Ru Ban, Xin Zhao, Hao Sun, Jingru Wang, Jie Lin, Tingting Guo, Jing Zhou, Zhangyong Xia

**Affiliations:** ^1^ Department of Neurology, Liaocheng People’s Hospital, Shandong University, Jinan, China; ^2^ Department of Neurology, Liaocheng People’s Hospital, Liaocheng, China; ^3^ Department of Neurology, The Third People’s Hospital of Liaocheng, Liaocheng, China; ^4^ Department of Neurology, Liaocheng People’s Hospital and Liaocheng Hospital Affiliated to Shandong First Medical University, Liaocheng, China; ^5^ School of Rehabilitation Medicine, Jining Medical University, Jining, China; ^6^ Department of Joint Laboratory for Translational Medicine Research, Liaocheng People’s Hospital, Liaocheng, China; ^7^ Department of Neurology, The First Affiliated Hospital of Shandong First Medical University & Shandong Provincial Qianfoshan Hospital, Jinan, China; ^8^ State Key Laboratory of Dampness Syndrome of Chinese Medicine, Shandong Sub-centre, Liaocheng, China; ^9^ Department of Neurology, The Second People’s Hospital of Liaocheng, Liaocheng, China

**Keywords:** carotid atherosclerosis, PAN-apoptosome related genes, diagnostic genes, bioinformatics, single-cell sequencing

## Abstract

**Background:**

Carotid atherosclerosis (CAS) is a complication of atherosclerosis (AS). PAN-optosome is an inflammatory programmed cell death pathway event regulated by the PAN-optosome complex. CAS’s PAN-optosome-related genes (PORGs) have yet to be studied. Hence, screening the PAN-optosome-related diagnostic genes for treating CAS was vital.

**Methods:**

We introduced transcriptome data to screen out differentially expressed genes (DEGs) in CAS. Subsequently, WGCNA analysis was utilized to mine module genes about PANoptosis score. We performed differential expression analysis (CAS samples *vs.* standard samples) to obtain CAS-related differentially expressed genes at the single-cell level. Venn diagram was executed to identify PAN-optosome-related differential genes (POR-DEGs) associated with CAS. Further, LASSO regression and RF algorithm were implemented to were executed to build a diagnostic model. We additionally performed immune infiltration and gene set enrichment analysis (GSEA) based on diagnostic genes. We verified the accuracy of the model genes by single-cell nuclear sequencing and RT-qPCR validation of clinical samples, as well as *in vitro* cellular experiments.

**Results:**

We identified 785 DEGs associated with CAS. Then, 4296 module genes about PANoptosis score were obtained. We obtained the 7365 and 1631 CAS-related DEGs at the single-cell level, respectively. 67 POR-DEGs were retained Venn diagram. Subsequently, 4 PAN-optosome-related diagnostic genes (*CNTN4*, *FILIP1*, *PHGDH*, and *TFPI2*) were identified via machine learning. Cellular function tests on four genes showed that these genes have essential roles in maintaining arterial cell viability and resisting cellular senescence.

**Conclusion:**

We obtained four PANoptosis-related diagnostic genes (*CNTN4*, *FILIP1*, *PHGDH*, and *TFPI2*) associated with CAS, laying a theoretical foundation for treating CAS.

## 1 Introduction

Atherosclerosis (AS) is a chronic inflammatory disease characterized by plaque accumulation in the intima of the arterial wall (1). Carotid atherosclerosis (CAS) is a symptomatic manifestation of AS at the carotid artery site, and the essential pathological change is the formation of plaques on the carotid artery intima, which are generally classified as lipid streaks, fibrous plaques, and atheromatous plaques (2). According to worldwide statistics, carotid atherosclerosis causes ischemic stroke, which can endanger patients’ lives (3). CAS is an independent vascular risk factor for cognitive dysfunction in non-stroke patients and can diminish patients’ cognitive function by impairing the structural and functional integrity of the brain (4). Due to its instability caused by features such as large plaque necrotic nuclei, thin fibrous cap, and speckle calcification with increased inflammation, rupture misses the early recognition stage. It leads to local thrombus formation (5). Currently, the diagnosis of CAS is mainly dependent on imaging and ultrasound, and no definitive and effective diagnostic biomarkers have been identified for CAS (6)

Cell death, apoptosis, and necrosis are genetic pathways of programmed cell death (PCD), which are involved in different ways in maintaining internal environmental homeostasis and developing diseases (7). Widespread apoptosis (PANoptosis), an inflammatory PCD pathway regulated by the PANoptosome complex, is crosstalk and overlaps the above three pathways (8). It has been shown that changes in the concentration of the pro-apoptotic molecules are associated with the development of CAS (9). Experimental studies have found that VX-765, an inhibitor of caspase-1, an important factor in cellular pyroptosis, may inhibit the process of Vascular smooth muscle cells (VSMCs) pyroptosis in CAS, which may be a potential therapeutic strategy for atherosclerotic diseases (10). However, the role of pan-apoptosis-related genes (PAN-optosome-related genes, PORGs) in CAS has yet to be reported.

In the era of bioinformatics, many joint analyses based on transcriptome sequencing (RNA-seq) and single-cell RNA sequencing (scRNA-seq) have been widely used in many studies as a powerful tool for the discovery of important cell types and diagnostic markers to identify relevant biomarkers for characterization (11). This joint analysis provides a more reliable theoretical basis for the study of disease pathogenesis, permitting the precise exploration of individual cell populations with unprecedented resolution (12).

In this study, we screened pan-apoptosis-related biomarkers in CAS based on the GEO database, combined with single-cell analysis tools, explored the biological processes in which they may be involved, and provided some implications for the clinical treatment of CAS patients.

## 2 Materials and methods

### 2.1 Data acquisition

The GSE100927 dataset collected from Gene Expression Omnibus (GEO) included 12 normal tissue samples and 29 CAS samples (**Supplementary Table S1**). The GSE43292 dataset comprised 32 CAS samples and 32 normal tissue samples (**Supplementary Table S2**). The single-cell dataset GSE159677 included 3 CAS patients with calcified atherosclerotic core (AC) plaques and three patient-matched proximal adjacent (PA) portions of the carotid artery.14 PAN-optosome related genes (PORGs)(**Supplementary Table S3**) were derived from previous research (7). The details of the selected datasets are presented in **Table 1**.

**Table 1:** The information of selected datasets in this study.

ID	Total sample number	CAS	control
GSE100927	41	29	12
GSE43292	64	32	32
GSE159677	6	3	3
CNP0004204	4	3	1

### 2.2 Single-nucleus RNA sequencing

#### 2.2.1 Human specimens and clinical characteristics

Carotid tissue samples for single-cell nuclear sequencing were obtained from three patients diagnosed with Carotid atherosclerosis and 44-year-old men diagnosed without Carotid atherosclerosis as the health control(**Table 2**). Consent was obtained from patients or their families before collecting all the samples used in this study. The Liaocheng City People’s Hospital Ethics Committee reviewed and approved this study (Ethics number:2019367). The single-cell sequencing data of the collected samples have been deposited in CNGBdb’s CNSA (CNGB Sequence Archive) (https://db.cngb.Org/cnsa/) under the accession number CNP0004204.

**Table 2:** Clinical features of single-cell sequencing.

Age (years)	gender	Diagnosis
53	male	Carotid atherosclerosis
59	female	Carotid atherosclerosis
68	male	Carotid atherosclerosis
44	male	Health control

#### 2.2.2 Cell nuclei isolation and snRNA-seq on a 10× genomics platform

Carotid plaque tissues were rinsed with 1× PBS and frozen. Cell nuclei were isolated via mechanical extraction, following the reference method (11). Tissues were thawed in a homogenizing buffer (20 mM Tris pH 8.0, 500 mM sucrose, 0.1% NP-40, 0.2 U/mL RNase inhibitor, protease inhibitor mixture, 1% BSA, 0.1 mM dithiothreitol), then ground using Dounce Pestle A and B, and filtered through 30 μm cell filters. The supernatant was centrifuged (500 × g, 5 min, 4°C), decanted, resuspended in 1× PBS with 1% BSA and 0.2 U/μL RNase inhibitor, and centrifuged again. The University of Washington’s ten × Genomics Chromium Single Cell 3′ kit was used for single-cell sequencing libraries. The 10X Chromium system created emulsions (GEMs) and gel beads with cell barcodes. A Thermo Fisher Scientific thermocycler aided GEM reverse transcription (53°C for 45 min, 85°C for 5 min, then cooled to 4°C) and cDNA amplification (98°C for 3 min; 12 cycles: 98°C for 15 s, 67°C for 20 s, 72°C for 1 min; final extension at 72°C for 1 min, then cooled to 4°C). Library construction followed cDNA purification using Beckman Coulter’s SPRIselect kit. Sequencing utilized the BGISEQ-500 system from BGI Group in collaboration with Welltec Biotechnology Co., Ltd., China.

### 2.3 Analysis of differential genes

To detect DEGs1 between regular and CAS groups in the GSE100927 dataset, the ‘limma’ software (13) was used. The threshold was set at *p.adj* < 0.05 and |log2FC| > 1. This ggplot2 volcano plot showed DEGs (DOI:10.18637/jss.v077.b02). Heatmapping revealed the top 20 DEGs (top 10 down-regulated and top 10 up-regulated).

### 2.4 Weighted gene co-expression network analysis

The ‘GSVA’ software calculated the ssGSEA score of 14 PORGs. SsGSEA scores were clinical characteristics. We grouped samples and deleted outliers to guarantee analytical accuracy. The trait heatmap, sample dendrogram, and soft threshold were then created. The phylogenetic tree was generated using gene similarity and adjacency. Gene modules with the highest correlation with clinical traits (|cor|=0.94 and *p*<0.01) were used as crucial for subsequent analysis.

### 2.5 Single-cell data processing

GSE159677 and our own scRNA-seq data. Those cells containing < 200 UMI (unique molecular identifier) and < 3 UMI were discarded. We performed the ‘Find-Variable Features’ function to screen the top 2000 highly variable genes for principal component analysis (PCA) analysis. Cells were clustered using consensus clustering and visualized with the UMAP algorithm based on the principal component. Clusters were annotated manually using the ‘SingleR’ package.

### 2.6 Differential gene expression of the single-cell and gene functional enrichment analysis

A differential gene expression analysis was conducted using clustered pairwise data (CAS samples *vs.* standard samples) using the Seurat FindMarker function. Gene Ontology (GO) and Kyoto Encyclopedia of Genes and Genomes (KEGG) enrichment analysis was conducted via ‘clusterProfiler package (14). *P value* < 0.05 was selected as the criteria.

### 2.7 Machine learning methods

Least absolute shrinkage and selection operator (LASSO) regression analysis (using the ‘glmnet’ package) and RF (using the ‘randomForest’ package) algorithm were applied to screen essential genes in the GSE100927 dataset. Diagnostic genes were obtained through taking the intersection genes of two machine learning algorithms. Then we used diagnostic genes to construct a diagnostic model associated with CAS. Moreover, the receiver operating characteristic (ROC) curve was plotted to evaluate the model’s value using the ‘pROC’ package (14). PCA was utilized to assess the difference between CAS and normal samples. At the same time, the GSE43292 was regarded as an external verification set for the diagnostic model.

### 2.8 Clinical nomogram model

The nomogram containing diagnostic genes was drawn via ‘rms’ to predict the risk CAS patients. The calibration curve evaluated the predictive effect. Finally, we draw ROC and decision curve analysis (DCA) curves to verify the calibration line.

### 2.9 Immune feature and GSEA analysis

The CIBERSORT algorithm was applied to calculate the relative abundance of 22 immune cells infiltrated in the CAS microenvironment. Subsequently, the correlation between diagnostic genes and differential immune cells was calculated and displayed. In addition, GSEA was conducted to explore the potential KEGG pathways associated with diagnostic genes through the ‘clusterProfiler’ package (15).

### 2.10 RNA extraction and validation of hub genes by qRT-PCR

A Steady Pure Quick RNA Extraction Kit (AG201023, AG, CHINA) was used for total RNA of carotid tissues of carotid atherosclerosis patients and health controls; Reverse transcription was conducted subsequently by a reverse transcription kit (AG11706, AG, CHINA). Amplification of cDNA was performed by utilizing the SYBR premixed Ex Taq kit (AG11718, AG, CHINA), with Glyceraldehyde 3-phosphate dehydrogenase as the internal reference. The 2(-Delta CT) method calculated the expression of mRNA levels. All the primers applied in the study are listed in **Table 3**.

**Table 3:** Primers used for RT-PCR in this study.

Gene	Forward primer(5′-3′)	Reverse primer(5′-3′)
GAPDH	TGCACCACCAACTGCTTAGC	GGCATGGACTG TGGTCATGAG
CNTN4	AACGCAGAGCTTAGTGTTATAGC	TTTGGAGACGCTT TTGGCTTA
FILIP1	GCCCTCCATCATCGGCAAT	GGTGTCGTTTGA CAGTTCCTG
PHGDH	CTGCGGAAAGTGCTCATCAGT	TGGCAGAGCGAA CAATAAGGC
TFPI2	CTGGGGCTGTCGATTCTGC	TCTCCGCGTTA TTTCCTGTTG

### 2.11 Cellular atherosclerosis model construction and cell transfection

Human aortic smooth muscle cells (HASMCs) were kindly provided by the Central Laboratory of Liaocheng People’s Hospital and cultured in high-fat medium, which includes DMEM/F12 (gibico, USA) medium supplemented with 10% FBS (BI, USA), 1 µg/mL penicillin/streptomycin(Hyclone, USA) and palmitate(PA, 500 μmol/L) at 37°C with 5% CO2 to imitate the high-fat milieu associated with atherosclerosis development.

### 2.12 Cell transfection

Full-length CNTN4, FILIP1, PHGDH, and TFPI2 were inserted into Ubi-MCS-3FLAG-SV40-IRES-puromycin Lentiviral Expression Vector and utilized for transfection of HASMC, and qRT-PCR investigated the impact of transfection.

### 2.13 Cell senescence and cell viability analysis

Human aortic smooth muscle cells (HASMC) with a density of 70% were treated with hydrogen peroxide (H_2_O_2_) at a concentration of 100 nM for 2 hours. The cells underwent a rinsing process using phosphate-buffered saline (PBS) followed by cultivation in a fresh medium for 24 hours. Cellular senescence was assessed using the Senescence β-Galactosidase Staining Kit (Biyun Tian, Shanghai, China).

A Live/Dead Cell kit (YEASEN, China) was applied to assess the cell viability of HASMC in the high-fat medium, and the fluorescence images were acquired by a fluorescence microscope (OLYMPUS, Japan).

### 2.14 Statistical analysis

The trials were conducted a minimum of three times. The statistical studies were conducted using R version 4.10 and GraphPad Prism version 8.0 software. The statistical distinctions between the groups were assessed by using a two-tailed Student’s t-test. The significance levels were denoted as follows: *, *p* < 0.05; **, *p* < 0.01; ***, *p* < 0.001.

## 3 Results

### 3.1 Identification of critical genes in CAS

**Figure 1:**
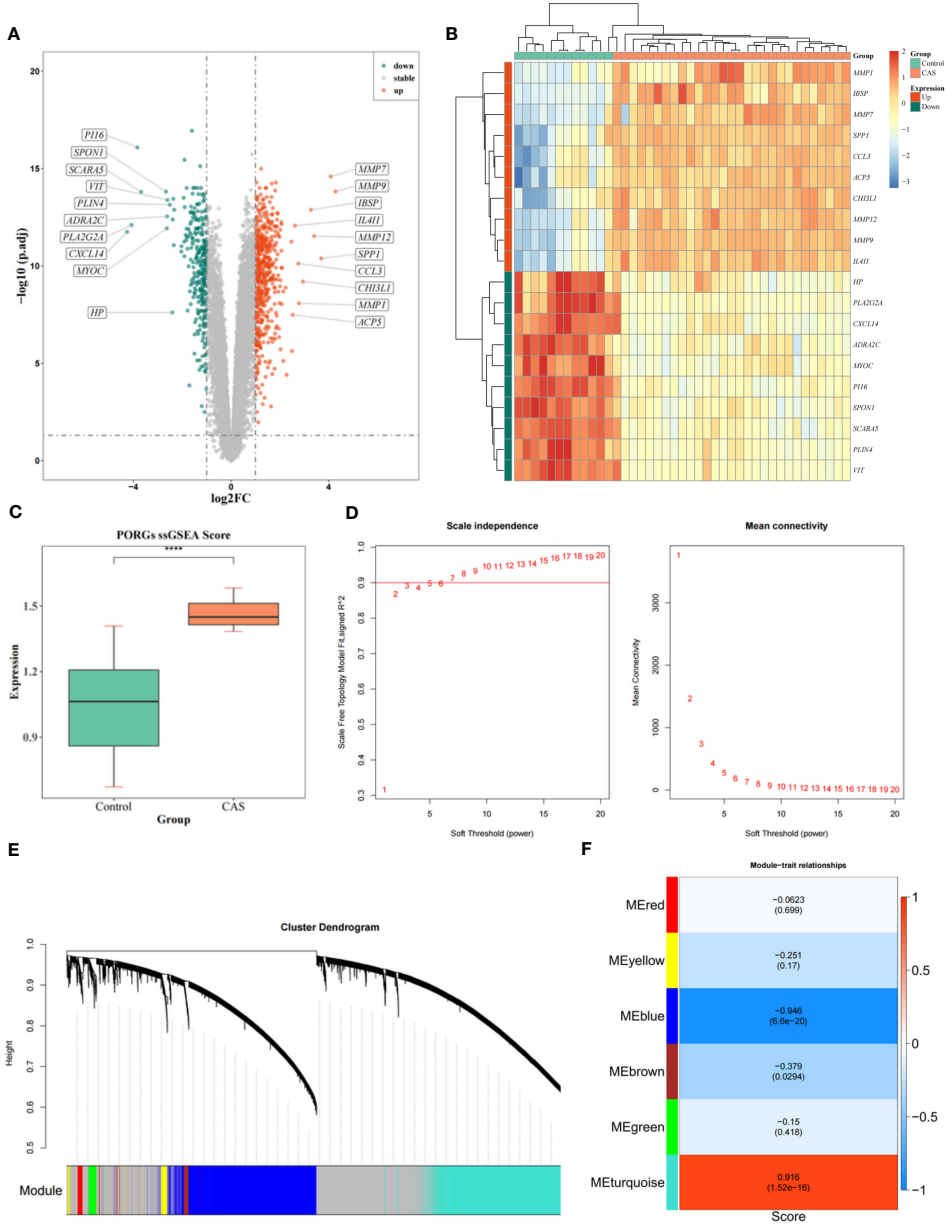
Identification of DEGs and WGCNA analysis. **(A)** Volcano map of DEGs. **(B)** Heatmap of DEGs. **(C)** Differences in PORGs scores in the CAS sample and control group. **(D)** The scale-free distribution was best captured by gene correlations when power = 7, according to soft threshold analysis. **(E)** The cluster dendrogram of co-expression in CAS. **(F)** Heatmap of correlations between different gene modules and traits, with p.adj values in parentheses and correlation coefficients outside parentheses for different modules.

785 DEGs1 were identified in CAS, including 284 down-regulated and 501 up-regulated genes (**Figure 1A**) (**Supplementary Table S4**). We selected the top 10 down-regulated and top 10 up-regulated genes to display in the heatmap (**Figure 1B**). To seek out pivotal modules related to CAS; we conducted a WGCNA. The scores of PORGs (CAS samples *vs.* standard samples) were found to be significantly different, so it was used as a clinical trait (**Figure 1C**) (**Supplementary Table S5**). The sample clustering results indicated no outlier samples (**Supplementary Figure 1**). The optimal soft threshold was 7. When mean connectivity was tended to 0, the ordinate scale-free fit index, signed R2, approached the threshold value of 0.85 (red line) (**Figure 1D**). A total of 7 modules were obtained by the dynamic tree cut algorithm (**Figure 1E**). The blue module was markedly correlated with scores of PORGs (**Figure 1F**) (**Supplementary Table S6**). Thus, 4296 essential module genes related to the PANoptosis score were obtained for subsequent analysis.

### 3.2 Single-cell atlas of CAS

**Figure 2:**
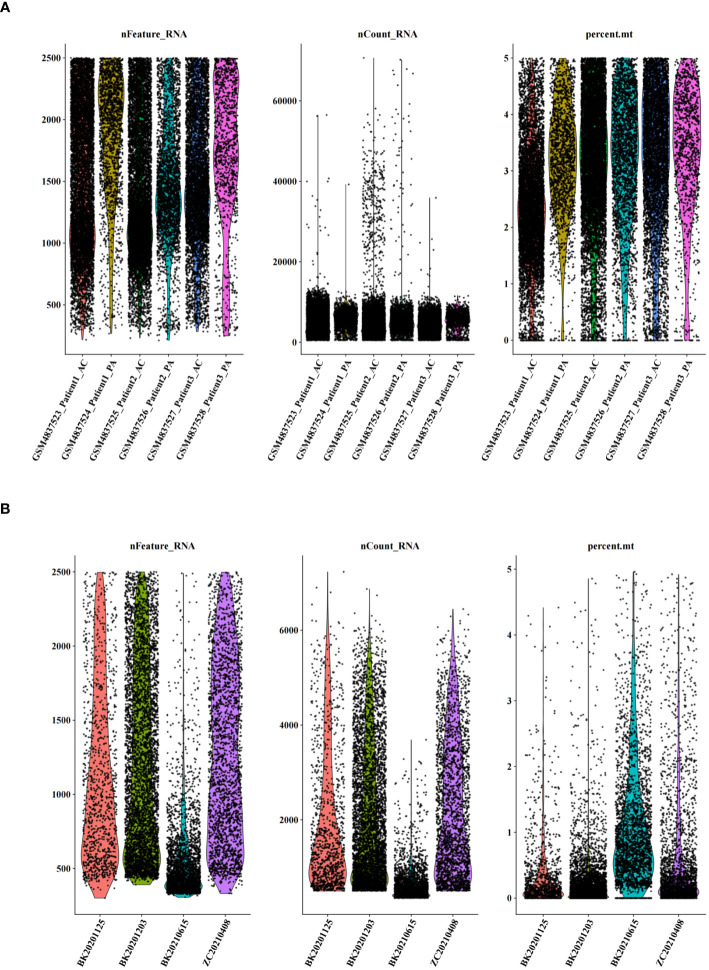
Quality control of single-cell data. **(A**) Number of genes, number of cells, and percentage of mitochondria sequenced for dataset GSE159677. **(B)** Number of genes, number of cells, and percentage of mitochondria sequenced for single-cell self-test data.

**Figure 3:**
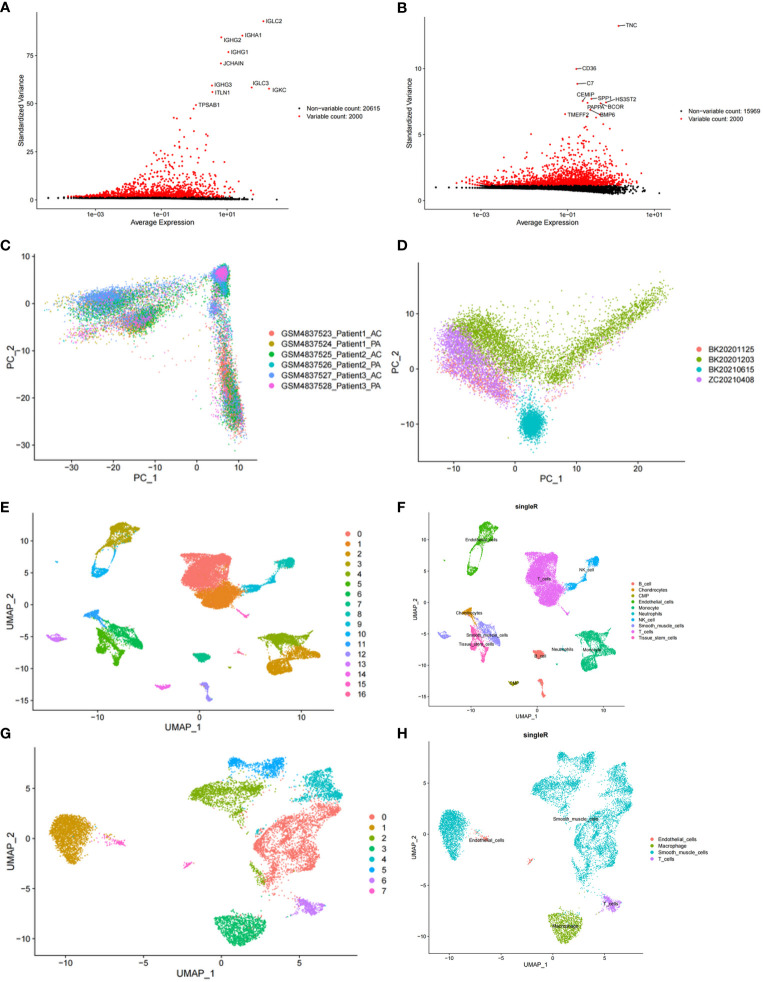
Clustering and annotation of self-test data and GSE159677 dataset. **(A)** 2000 highly variable genes in self-assay data. **(B)** 2000 highly variable genes in the GSE159677 dataset. **(C, D)** PCA plots of single-cell data. **(E, F)** Dataset GSE159677 is divided into 16 clusters and 9 cell types. **(G, H)** Self-test data is divided into 7 clusters and 4 cell types.

After cell filtration, we performed cell clustering and annotation analysis on GSE159677 and self-test datasets (**Figures 2A, B**). In total, 2000 highly variable genes were identified (**Figures 3A, B**). PCA plots revealed that the P values of 50 principal components were highly significant (**Figures 3C, D**). Therefore, we chose these 50 main components for subsequent analysis. In the GSE159677 dataset, we found that the cells were divided into 16 clusters (**Figure 3E**), which were annotated as T cells, Monocyte, Endothelial cells, Tissue stem cells, Smooth muscle cells, B cells, NK cells, Chondrocytes, CMP, and Neutrophils (**Figure 3E**) (**Supplementary Table S7**). Meanwhile, the cells of the self-test were divided into 7 clusters (**Figure 3F**), which were annotated as T cells, Endothelial cells, Smooth muscle cells, and Macrophage (**Figure 3F**) (**Supplementary Table S8**). Three cells were co-annotated: smooth muscle cells, Endothelial cells, and T cells.

### 3.3 Identification of DEGs in CAS

**Figure 4:**
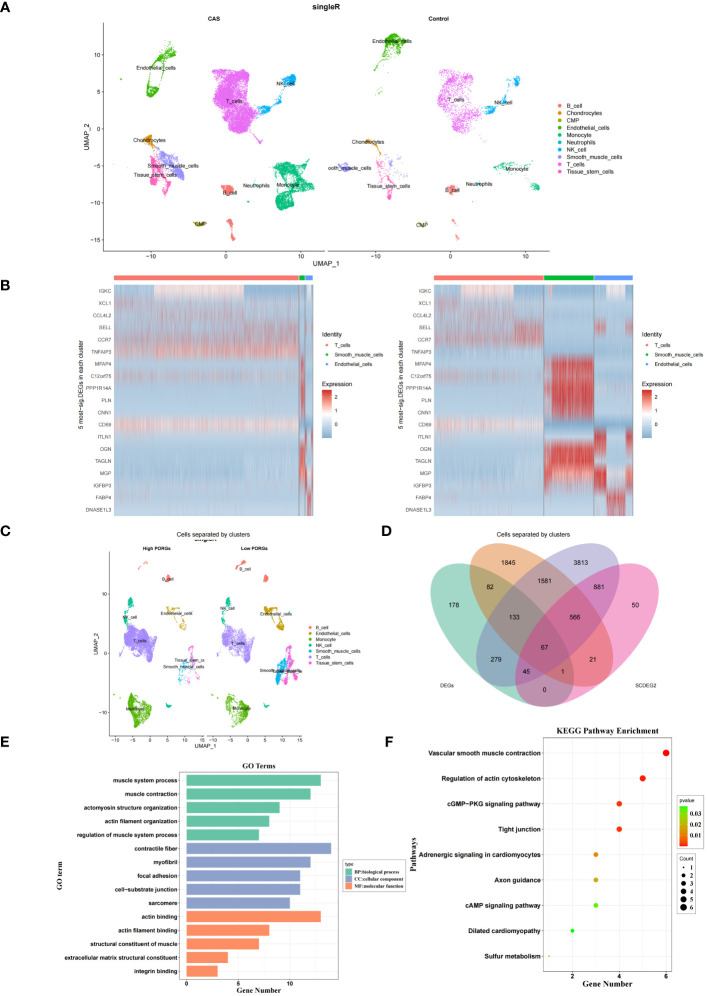
DEGs in CAS. **(A)** Annotation of CAS and normal cells in the GSE159677 dataset. **(B)** Heatmap of pan-apoptotic gene high expression group vs. low expression group. **(C)** Single-cell annotation of pan-apoptotic gene high-expression and low-expression groups. **(D)** Intersecting genes of module genes, ScDEGs1, ScDEGs2, and training set differential genes. **(E, F)** Enrichment analysis of GO **(E)** and KEGG **(F)**. DEGs, differentially expressed genes; CAS, carotid atherosclerosis; GO, Gene Ontology; KEGG, Kyoto Encyclopedia of Genes and Genomes.

In the GSE159677 dataset, 7365 ScDEGs1 between CAS and standard samples were identified at the single-cell level. We performed separate cellular annotations for CAS and regular groups in the GSE159677 dataset (**Figure 4A**) (**Supplementary Table S9**). We calculated the expression ratio of 14 PORGs in the co-annotated cells associated with CAS samples. Difference analysis found that 1631 ScDEGs2 related to PANoptosis were identified between two expression groups (**Figure 4B**) (**Supplementary Table S10**). According to the median expression ratio, the cells were divided into a high expression group and a low expression group (**Figure 4C**). Similarly, Furthermore, 67 PAN-optosome related differential genes (POR-DEGs) associated with CAS were retained by training-set DEGs, essential module genes, ScDEGs1 and ScDEGs2 (**Figure 4D**) (**Supplementary Table S11**). We proceeded with functional enrichment analysis to uncover potential mechanisms for POR-DEGs associated with CAS. Accordingly, 257 GO items and 9 KEGG pathways were gained. The top 5 GO items under each classification are shown in (**Figure 4E**) (**Supplementary Table S12**). We observed that the above genes were principally linked to the ‘regulation of muscle system process’ and ‘actomyosin structure organization.’ In addition, the KEGG results showed that these genes were mainly enriched in the ‘cGMP−PKG signaling pathway’ and ‘cAMP signaling pathway’ (**Figure 4F**) (**Supplementary Table S13**).

### 3.4 Construction of PANoptosis-related diagnostic model for CAS

**Figure 5:**
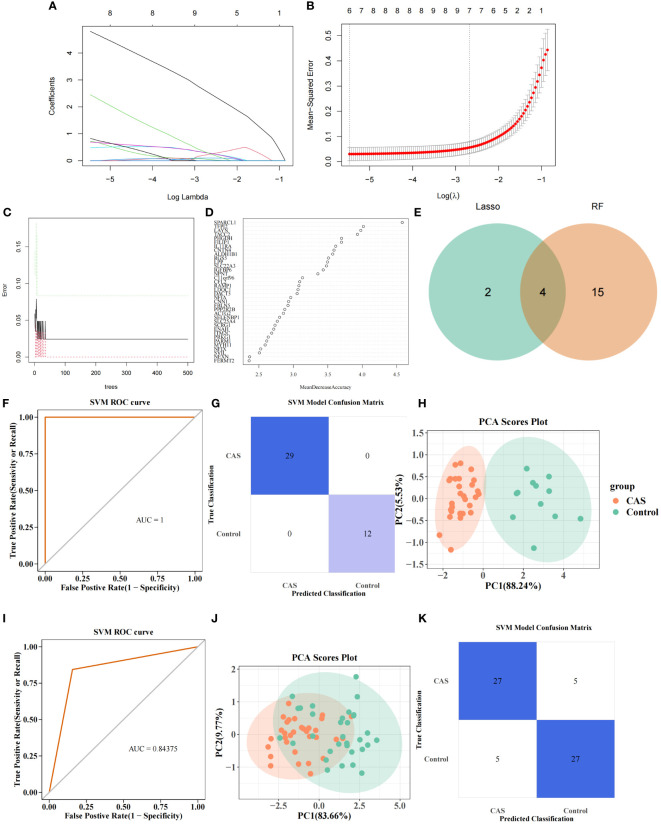
PANoptosis-related diagnostic model for CAS. **(A)** Gene coefficient maps for 67 genes. **(B)** LASSO regression analysis on 67 genes. **(C)** Number ofoptimal classification trees. **(D)** Importance ranking of characterization genes. **(E)** Intersecting genes screened by Lasso regression analysis and RF algorithm. **(F)** PANoptosis-related diagnostic model. **(G)** SVM confusion matrix. **(H)** PCA analysis of CAS and normal samples. **(I–K)** Diagnostic models in the validation set **(I)**, SVM confusion matrix **(J)**, and PCA analysis of CAS and normal samples **(K)**.

LASSO regression analysis was performed on 67 POR-DEGs to dig out the essential genes further to unearth the optima. Ultimately, 6 feature genes were obtained (**Figures 5A, B**). Meantime, 19 feature genes were retained by the RF algorithm (**Figures 5C, D**) (**Supplementary Table S14**). Subsequently, we combined 4 overlapping diagnostic genes between these two methods, including *CNTN4*, *FILIP1*, *PHGDH*, and *TFPI2* (**Figure 5E**). Therefore, a PANoptosis-related diagnostic model was build and evaluated in CAS. The AUC value was more significant than 0.7, indicating that the model had good accuracy (**Figure 5F**). PCA analysis and confusion matrix showed that the model could distinguish the CAS and control samples (**Figures 5G, H**). Next, we further validated the diagnostic model in the external validation set (GSE43292). We acquired consistent results with the GSE159677 (**Figures 5I–K**).

### 3.5 Construction and verification of nomogram

**Figure 6:**
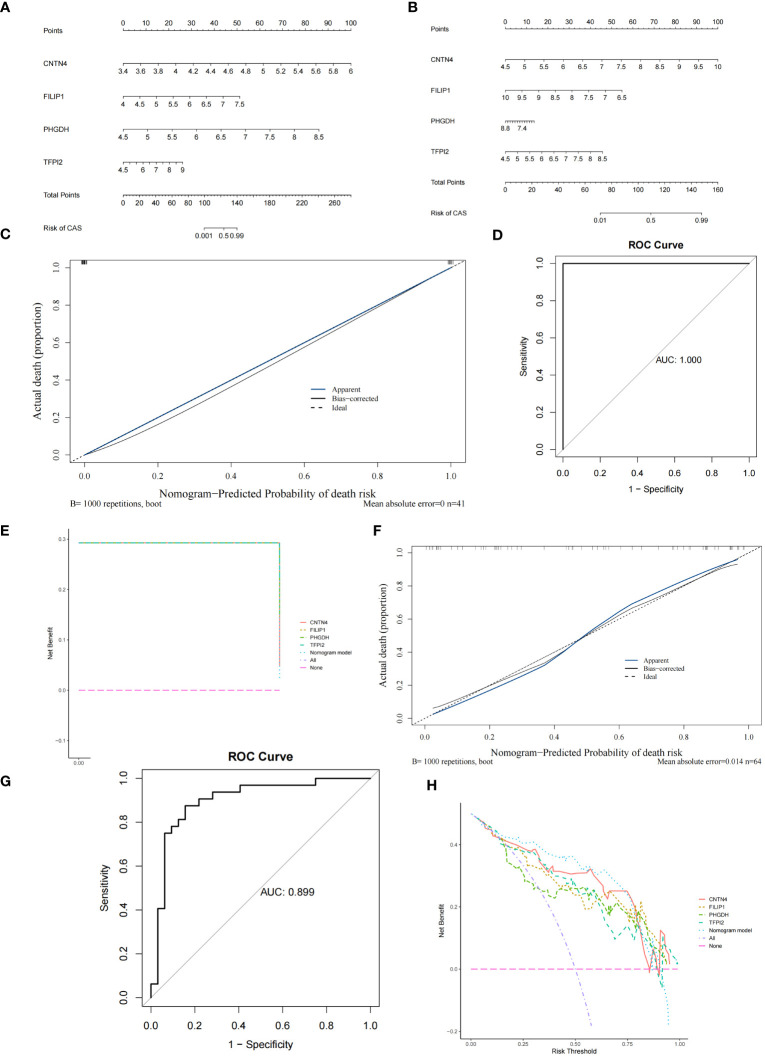
Construction and verification of Nomogram diagnostic model. **(A)** Nomogram of 4 diagnostic genes in GSE100927. **(B)** Nomogram of 4 diagnostic genes in GSE43292. **(C)** Calibration curve of GSE100927. **(D)** ROC curve of GSE100927. **(E)** DCA curve of GSE100927. **(F)** Calibration curve of GSE43292. **(G)** ROC curve of GSE43292. **(H)** DCA curve of GSE43292.

The nomogram containing 4 diagnostic genes was generated in GSE100927 and GSE43292, respectively, and total points could predict risk of CAS (**Figures 6A, B**) (**Supplementary Table S15**). The slopes of the calibration curves were all close to 1. Area under the ROC curve (AUC) values were 1.000 and 0.899 in GSE100927 and GSE43292, respectively. DCA curves showed that nomogram had higher net benefits than extreme curves. The calibration, ROC, and DCA curves proved that the performance of the PO-related CAS diagnostic model was effective (**Figures 6C–H**).

### 3.6 Immune infiltration and functional enrichment analysis

**Figure 7:**
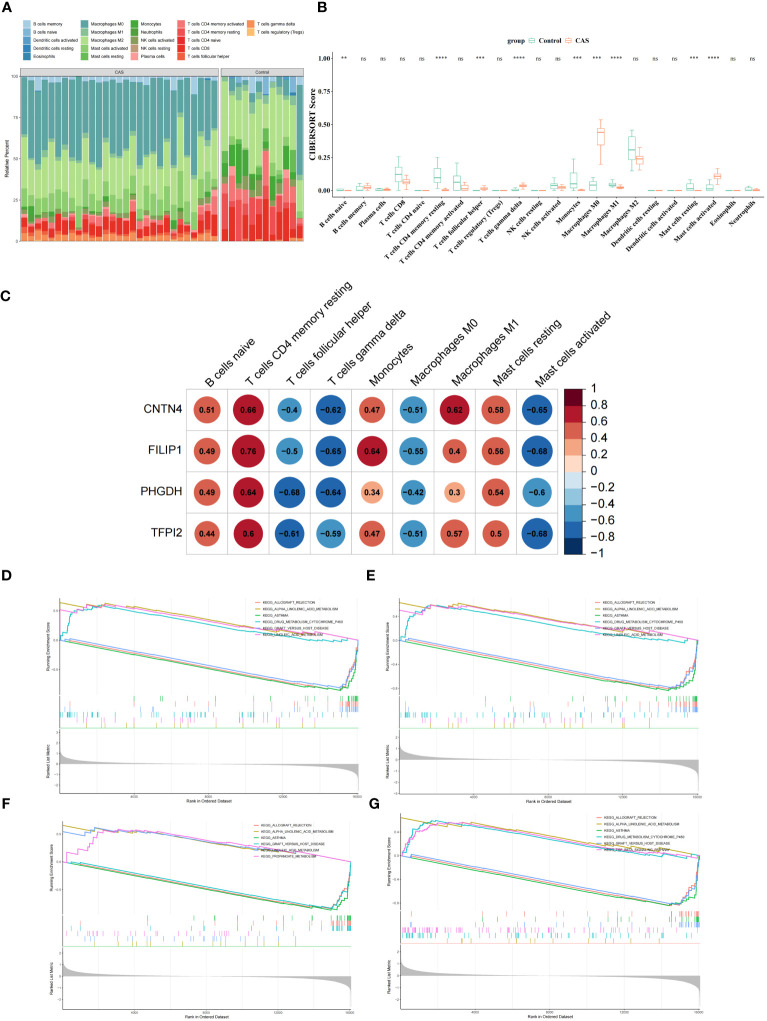
Immune microenvironment of CAS. **(A)** Comparison of CAS and normal samples using a heat map of the 22 immune cell subpopulations. **(B)** Expression of different immune cells in CAS and normal. **(C)** Heatmap of the correlation of CNTN4, FILIP1, PHGDH, and TFPI2 with different immune cells. **(D–G)** Single-gene GSEA of CNTN4 **(D)**, FILIP1 **(E)**, PHGDH **(F)** and TFPI2 **(G)**. ns *p* > 0.05, ** represents p less than 0.01, *** represents p less than 0.001, **** represents p less than 0.0001.

To explore the immune microenvironment of CAS, we showed the abundance of 22 immune cells between two sample groups in GSE100927 (**Figure 7A**) (**Supplementary Table S16**). Notably, 9 immune cell abundances differed significantly, including naive B cells, CD4 memory resting T cells, follicular helper T cells, Monocytes, M0 Macrophages, M1 Macrophages, resting mast cells, activated mast cells and gamma delta T cells (**Figure 7B**) (**Supplementary Table S17**). The correlation analysis revealed that 4 diagnostic genes were associated with significantly different immune cells (**Figure 7C**) (**Supplementary Table S18**). To further study the potential roles of *CNTN4*, *FILIP1*, *PHGDH*, and *TFPI2* in CAS, we performed single-gene GSEA on diagnostic genes. KEGG results showed that the ‘allograft rejection’ pathway was enriched in the groups with low-expression of *CNTN4*, *FILIP1*, *PHGDH*, and *TFPI2*. In contrast, ‘alpha linolenic acid metabolism’ and ‘asthma’ pathway were associated with high-expression groups (**Figures 7D–G**) (**Supplementary Table S19**). The results of GO suggested that the ‘cGMP metabolic process’ and ‘elastic fiber assembly process were enriched in the groups with high expression of *CNTN4*, *FILIP1*, *PHGDH*, and *TFPI2* (**Supplementary Figure 2**).

### 3.7 Expression validation of the diagnostic genes

**Figure 8:**
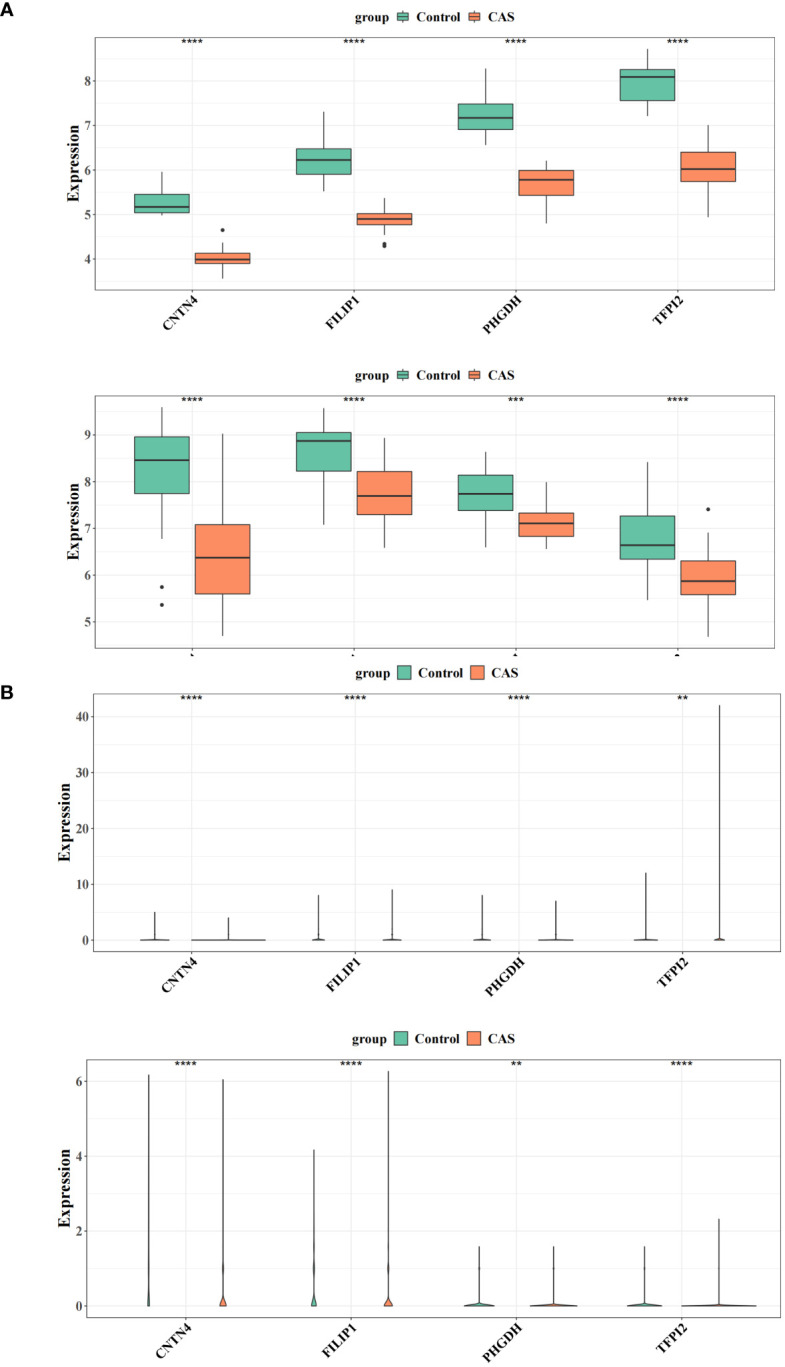
Expression validation of the diagnostic genes. **(A)** Differences in the expression of four genes in the training and validation sets of CAS versus the normal group. **(B)** Differential expression of four genes in the single-cell dataset GSE159677 and the self-test dataset. ** represents p value less than 0.01, *** represents p less than 0.001, **** represents p less than 0.0001.

At the transcriptome level, the expression of diagnostic genes was analyzed in the GSE100927 and GSE43292, respectively. We observed higher expression of *CNTN4*, *FILIP1*, *PHGDH*, and *TFPI2* in a regular group compared to the CAS group (**Figure 8A**) (**Supplementary Table S20**). At the single-cell level, the expression of diagnostic genes was analyzed in the GSE159677 and self-test datasets, respectively (**Figure 8B**) (**Supplementary Table S21**). The results suggested that the differential expression of diagnostic genes was remarkable.

### 3.8 RNA extraction and validation of hub genes by qRT-PCR

**Figure 9:**
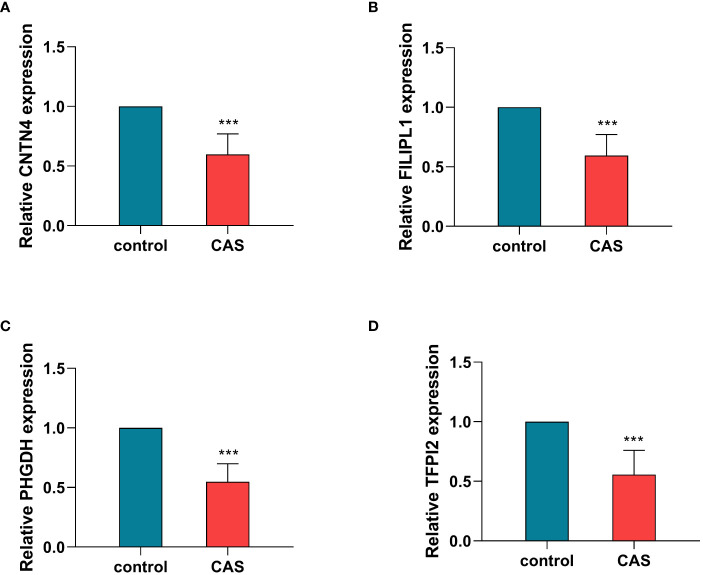
Validation of hub genes by qRT-PCR. The relative expression levels of CNTN4 **(A)**, FILIPLI **(B)**, FHGDH **(C)**, and TFPI2 **(D)** in control and carotid atherosclerosis samples identified by RT-PCR, with GAPDH as a reference, ***P < 0.001. CAS, carotid atherosclerosis.

The qRT - PCR results were consistent with our analysis that the expression of CNTN4, FILIP1, PHGDH, and TFPI2 (**Figures 9A–D**) (**Supplementary Table S22**) in plaques was significantly lower than in controls.

### 3.9 Cell senescence and cell viability analysis

**Figure 10:**
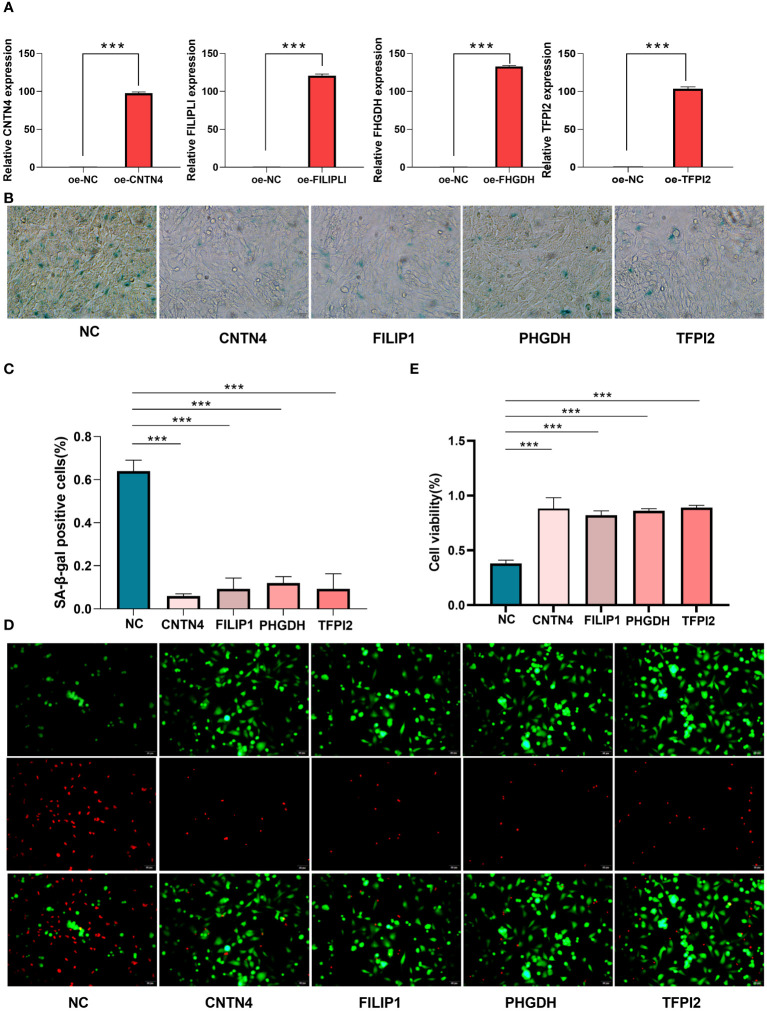
Cell senescence and cell viability analysis of HASMCs. **(A)** qPT-PCR of over-expression of CNTN4, FILIP1, PHGDH, and TFPI2; **(B, C)** SA-β-Gal staining showed the over-expression of CNTN4, FILIP1, PHGDH and TFPI2 reduced senescence of HASMC treated with H_2_O_2_; **(D, E)** Live/Dead staining showed that CNTN4, FILIP1, PHGDH, and TFPI2 increased cell viability of HASMC in a high lipid damage environment compared to the blank control groups. *** represents p less than 0.001.

For cellular functions, the qRT - PCR showed high transfection efficiency of CNTN4, FILIP1, PHGDH, and TFPI2 (**Figure 10A**) (**Supplementary Table S23**).

β-Galactosidase staining showed that these four genes reduced the cell senescence of HASMC(**Figures 10B, C**) (**Supplementary Table S24**) and the live/dead staining results and maintained higher cell viability in a high lipid environment compared to the blank control group (**Figures 10D, E**).

## 4 Discussion

Increasing evidence suggests that PORGs are essential in natural immune cells and can influence the developmental processes of infectious diseases, autoimmunity, and cancer (16). In this study, we identified 67 POR-DEGs and constructed a new diagnostic model about CAS consisting of four POR-DEGs. We mined and collected 22 clinical samples to validate four gene expression profiles through self-assay data from single-cell sequencing in conjunction with public databases. For cellular experiments, we used human aortic smooth muscle cells to construct a high-fat injury model, and cell viability assays and cell senescence assays confirmed our previous analyses. In addition, we explored possible mechanisms of action of diagnostic biomarkers involved in the CAS immune microenvironment. In addition, we explored the potential mechanisms of action of diagnostic biomarkers involved in the immune microenvironment of CAS.

CNTN4 (Contactin-4) is a neuronal cell adhesion molecule encoding an immunoglobulin superfamily (IgCAMs) (17). CNTN4 is ubiquitously expressed in most GABAergic interneurons and glutamatergic pyramidal neuron populations. CNTN4 has been shown to promote synaptic elongation by interacting with Ptprg, thereby promoting synaptic extension (18). ZUKO et al. found that the knockdown of CNTN4 in mice resulted in the production of severe phenotypes that were dissimilar to those of the normals, suggesting that CNTN4 may go on to promote the development of the brain in a region-specific manner Contactins: structural aspects about developmental functions in brain disease.

In the present study, CNTN4 was lowly expressed in the CAS group compared with the standard group, and we conjectured that this low expression might affect the synapses and thus produce brain hypoplasia in CAS patients.

FILIP1 belongs to a kind of Filamin A-interacting protein (FILIP) (19). The literature has reported that FILIP regulates the process of cell shape dynamics in neocortical cell migration from the ventricular zone through the filamin A-F-actin axis (19). Deletion of 6q14 has been associated with intellectual disability and several significant micro deformities, and FILIP1 has been suggested to be one of the potential genes in 6q14 that may be related to intellectual disability. We found that FILIP1 was differentially expressed in CAS and regular groups, possibly related to cognitive dysfunction in CAS patients.

Phosphoglycerate dehydrogenase (PHGDH) is a dehydrogenating metabolizing enzyme involved in the serine synthesis pathway (SSP), which has been found to play a central role in cancer growth and proliferation (20). A deficiency of PHGDH causes psychomotor developmental delay (21). Experiments in rodents have found that silencing of PHGDH expression results in the loss of L-serine synthesis in neurons after terminal differentiation, leading to severe neurological symptoms (22). Its low expression in the CAS group may have some relation to the neurological damage causing the disease.

TFPI-2 (Tissue factor pathway inhibitor-2) is a member of the Kunitz-type serine protease inhibitor family and is a structural homolog of tissue factor pathway inhibitor (TFPI) (23). Several studies have found that TFPI-2 plays a role in maintaining the stability of the tumor microenvironment and is considered a promising prognostic biomarker in several cancers (24). In this study, we found for the first time that TFPI-2 was significantly under-expressed in CAS, which suggests that it is likely to play a vital role in the mechanism of CAS and deserves to be further investigated in depth.

Our analysis revealed that CNTN4, FILIP1, PHGDH, and TFPI2 were co-enriched in the Alpha-linolenic acid metabolism pathway. Pharmacological studies have shown that alpha-linolenic acid (ALA) is an essential fatty acid in the human body with anti-metabolic syndrome and neuroprotective effects. It has also been found that high levels of ALA are associated with the development of cardiovascular disease (CVD) (25). Therefore, it is reasonable to assume that these four key biomarkers can influence the occurrence of CAS by participating in this biological pathway. Similarly, the ‘cGMP metabolic process’ is a typical physical process for all four biomarkers. Cyclic guanosine monophosphate (cGMP) is a second messenger molecule formed in different cell types and tissues (26). cGMP metabolic process regulates the cardiovascular system and brain function and participates in the pathogenesis of neurodegenerative diseases (27) controls the cardiovascular system and brain function. In CAS, these four markers will likely influence the condition by playing a role in this biological process.

It is widely accepted that CAS is caused by an imbalance between pathogenic T cells and Treg (regulatory T cells) (28). The balance of immune cells and their interaction with inflammatory cells have been found to contribute to the formation and maintenance of atherosclerotic plaques (29). CAS can cause pathological changes in the infiltration of immune cells (30)]. Ali et al. suggested that CAS produces several metabolic changes and that monocyte and macrophage function is critical for metabolic changes (31). In our study, we found that there were nine types of immune cells, including CD4 memory resting T cells, Monocytes, M0 Macrophages, and M1 Macrophages, with significant differences in levels between the CAS group and the standard group, which reinforces the conclusions of the authors mentioned above on the role of immune cells in CAS.

Single-cell analysis technology has been widely used in basic and clinical research, essential for the early diagnosis, tracking, and individualized treatment of diseases. In this study, we screened three cell clusters at the single-cell level that may have essential roles in CAS, including vascular smooth muscle cells, Endothelial cells, and T cells. VSMCs are cells in atherosclerotic plaques. A significant cell type is present in all stages of atherosclerotic plaques, and their abnormal proliferation promotes plaque formation (32). Endothelial cells (ESCs) are an integral part of the heart and blood vessels and have a direct link between the cardiovascular and immune systems (33). It has been reported in the literature that Endothelial cells may go on to participate in the CAS process through complex signaling mechanisms such as cellular pyroptosis, apoptosis, and autophagy (34). CAS plaques are activated to release inflammatory molecules through the infiltration of T cells and plasma cells, among others, to manifest symptoms (35, 36). Thus, these three key cell clusters provide strong evidence for our subsequent in-depth mechanistic studies of CAS at the cellular level.

In conclusion, we identified four potential biomarkers (CNTN4, FILIP1, PHGDH, and TFPI2). We established a pan-apoptosis-related CAS diagnostic model through the combined bioinformatics analysis of CAS disease-related microarray expression profiling data and single-cell data, which provided new insights and references for the mechanistic study of CAS disease. Even though we conducted PCR in 11 carotid plaques versus 11 normal arterial vessels and experimentally validated four genes related to cell viability and cellular senescence at the cellular level, a more in-depth mechanistic study is required, which will be elaborated in our subsequent studies. Although we performed gene function experiments on the four screened genes, larger clinical samples for testing and next steps for animal experiments are still needed.

## Data availability statement

The data presented in the study are deposited in the NCBI repository (https://www.ncbi.nlm.nih.gov/), accession number GSE100927, GSE43292 and GSE159677; and CNGBdb repository (https://db.cngb.Org/cnsa/), accession number CNP0004204 and CNP0004860.

## Ethics statement

The present study received ethical approval from the Ethics Committee of Liaocheng People’s Hospital (Approval No. 2019367). The studies were conducted in accordance with the local legislation and institutional requirements. Written informed consent for participation in this study was provided by the participants’ legal guardians/next of kin. Written informed consent was obtained from the individual(s) for the publication of any potentially identifiable images or data included in this article.

## Author contributions

YS: Writing – original draft, Writing – review & editing. BL: Writing – original draft, Writing – review & editing. HW: Writing – original draft, Writing – review & editing. GZ: Writing – original draft, Writing – review & editing. YX: Writing – original draft, Writing – review & editing. RB: Writing – original draft, Writing – review & editing. XZ: Writing – original draft, Writing – review & editing. HS: Writing – original draft, Writing – review & editing. JW: Writing – original draft, Writing – review & editing. JL: Writing – original draft, Writing – review & editing. TG: Writing – original draft, Writing – review & editing. JZ: Writing – original draft, Writing – review & editing. ZX: Writing – original draft, Writing – review & editing.
